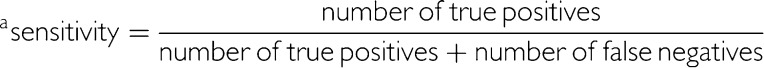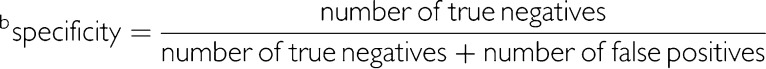# Trefoil factor 3: a novel serum marker identified by gene expression profiling in high-grade endometrial carcinomas

**DOI:** 10.1038/sj.bjc.6604546

**Published:** 2008-08-05

**Authors:** E Bignotti, A Ravaggi, R A Tassi, S Calza, E Rossi, M Falchetti, C Romani, E Bandiera, F E Odicino, S Pecorelli, A D Santin

**Affiliations:** 1 Department of Obstetrics and Gynecology, Division of Gynecologic Oncology, University of Brescia, Viale Europa 11, 25123 Brescia, Italy; 2 Section of Medical Statistics and Biometry, Department of Biomedical Sciences and Biotechnology, University of Brescia, Viale Europa 11, 25123 Brescia, Italy; 3 Department of Pathology, University of Brescia, Viale Europa 11, 25123 Brescia, Italy; 4 Department of Obstetrics, Gynecology and Reproductive Sciences, Yale University School of Medicine, 333 Cedar Street, PO Box 208063, New Haven, CT 06520-8063, USA

**Keywords:** G3 endometrioid endometrial carcinoma, microarray technology, trefoil factor, serum marker, sandwich ELISA

## Abstract

This study identifies the genetic fingerprint of poorly differentiated endometrioid endometrial carcinomas (G3-EEC) and analyses the potential utility of trefoil factor 3 (TFF3) as novel serum marker in G3-EEC. Affymetrix microarrays were used to identify the gene expression patterns of 19 snap-frozen G3-EEC and 15 normal endometrium (NE) biopsies. Quantitative real-time PCR (qRT-PCR) and immunohistochemistry were used to validate TFF3 expression. Finally, TFF3 serum levels were determined by ELISA in 25 G3-EEC patients, 42 healthy controls, and in 13 endometrial hyperplasia patients. Hierarchical cluster analysis showed TFF3 as the top differentially expressed gene between 363 upregulated genes in G3-EEC, when compared with NE. Trefoil factor 3 gene expression levels analysed by qRT-PCR significantly correlated with Affymetrix results (*P*<0.001; rs=0.85). By immunohistochemistry, TFF3 protein was significatively more expressed in EEC compared with NE (*P*<0.01), with cytoplasmatic positivity in 79% G3-EEC and 18% NE. Patients harbouring G3-EECs had significantly higher TFF3 serum concentration by ELISA when compared with healthy patients (*P*<0.001) or patients harbouring endometrial hyperplasia (*P*=0.012). In conclusion, TFF3 is highly expressed at gene and protein level in G3-EEC. Further investigations on a wider set of samples are warranted to validate TFF3 as a novel serum marker for early detection and/or monitoring of G3-EEC patients.

Endometrial carcinoma (EC) is the most common gynecologic malignancy in Western world and it is characterized by two clinical/pathogenic types (Bokhman, 1983). Type I ECs, which account for the majority of cases, are oestrogen-related tumours usually well differentiated and endometrioid in histology. Typically these patients have a favourable prognosis with appropriate therapy. In contrast, Type II ECs include poorly differentiated endometrioid endometrial tumours (G3-EEC), serous papillary, and clear cell ECs. These tumours are not associated with hyperoestrogenic factors, and they are more likely to be deeply invasive in the myometrium and/or metastatic at presentation and often recur despite aggressive clinical interventions. Poorly differentiated endometrioid endometrial carcinomas account for the majority of Type II Ecs, and unfortunately, to date, no good marker for screening or disease monitoring for these biologically aggressive cancers is available. In this regard, CA125 is often used in clinical practise to monitor EC patients (Duk *et al*, 1986). However, this marker appears to have limited utility in monitoring the effects of adjuvant therapy or in the prediction of tumour recurrence (Chung *et al*, 2006).

Large-scale gene expression analysis using microarrays represents a powerful tool to discover gene expression patterns characteristic for different human tumours. Consistent with this view, in the last few years, several investigators have used this technology in an attempt to identify gene expression profiling characteristic of ECs and its different histological subtypes (Risinger *et al*, 2003; Planagumà *et al*, 2004). However, although the degree of histologic differentiation in EEC patients has long being accepted as the most sensitive indicator of prognosis, to our knowledge, no studies have investigated the genetic fingerprint of G3-EEC separately from those of G1 and G2-EEC and/or the other histologic variants of Type II EC (i.e., serous papillary and clear cell tumours).

In this study, we have carefully analysed the gene expression pattern of 19 G3-EECs and 15 normal endometria (NEs) using oligonucleotide microarrays with probe sets complementary to 38 500 well-characterized human genes. Among the 363 genes upregulated in EECs when compared with NEs, human intestinal trefoil factor 3 (TFF3, gene symbol hITF) was found as the top highly expressed gene in G3-EECs. In this regard, TFF3 belongs to a family of small mucin-associated polypeptides, mainly present in the gastrointestinal tract and other epithelial tissues, known to play an important function in maintaining mucosal integrity (Hoffmann *et al*, 2001). Recently, TFFs have been reported to be overexpressed at the gene and protein level in human neoplasms, including intestinal, pancreatic, and prostate cancers (Taupin *et al*, 1996; Terris *et al*, 2002; Garraway *et al*, 2004).

In this study, with the aim to investigate the potential utility of TFF3 in the diagnosis and/or monitoring of G3-EEC, we have validated its expression levels by quantitative real-time PCR, whereas protein expression was tested by immunohistochemistry. Furthermore, using a novel in-house made ELISA assay, we have measured preoperative TFF3 serum levels in patients with G3-EECs, with endometrial hyperplasia, and in healthy female controls. In addition, we have compared TFF3 serum levels with those of CA125, the marker more often used in EC clinical practise.

## Materials and methods

### Patients and samples

A total of 19 snap-frozen G3-EECs and 15 NE were collected from the Division of Gynecologic Oncology at the University of Brescia (Italy) from 2003 to 2006. Study approval was obtained from the Institutional Review Board, and all patients signed an informed consent according to institutional guidelines. Tumour tissues were obtained from women undergoing complete surgical staging, which included total abdominal hysterectomy, bilateral salpingo-oophorectomy, pelvic and periaortic lymphadenectomy, and peritoneal washings for cytology. All patients were staged in accordance with International Federation of Gynaecologists and Obstetricians (FIGO) guidelines. None of the patients had received preoperative chemotherapy or radiation. Moreover, samples of NE were collected from age-matched patients.

Tumour and normal tissues sharp-dissection, liquid nitrogen freezing, and epithelial purity checking were performed as previously reported (Bignotti *et al*, 2006).

Immunohistochemical analysis was performed on 38 G3-EECs and 22 NEs, collected in the Department of Pathology, University of Brescia, Italy.

Preoperative serum samples from 25 patients with G3-EEC, 13 patients with endometrial hyperplasia (EH), and 42 healthy female controls were stored. All serum samples were collected before any patient treatment, frozen in liquid nitrogen within 2 h of blood drawing, and stored at −80°C.

### Total RNA extraction and Genechip hybridisation

Total RNA extraction, quantification, and quality assessment were performed as previously described (Bignotti *et al*, 2006). Labelling of samples and hybridisation to the Affymetrix Human HG-U133 Plus 2.0 oligonucleotide microarray chips (Santa Clara, CA, USA), covering over 47 000 human transcripts and variants, were performed following the manufacturer's protocols.

### Quantitative real-time PCR

Quantitative real-time polymerase chain reaction (qRT-PCR) was performed in triplicate by using primer sets and probes specific for TFF3 gene (Assay on Demand Hs00173625_m1, Applied Biosystems, Foster City, CA, USA). Complementary DNA synthesis and PCR conditions were performed as previously described (Bignotti *et al*, 2006).

### Immunohistochemistry on formalin-fixed, paraffin-embedded tissues

Immunostaining of 38 formalin-fixed, paraffin-embedded G3-EEC and 22 NE tissues was performed as previously described (Bignotti *et al*, 2006), using the TFF3 mouse monoclonal antibody 1 *μ*g ml^−1^ (Assay Designs, Ann Arbor, MI, USA). All samples were scored quantitatively and qualitatively in 20 and 40 high-power fields in every section (Nikon, Tokio, Japan, Eclipse E400). Slides were blindly analysed by three independent pathologists, and the scoring method was based on the intensity of the staining and on the percentage of tumour cell stained. Intensity was scored as follows: 0 indicating no staining; 1 weak staining; 2 moderate staining; and 3 strong staining. The percentage of tumour cells stained was scored as follows: 0 indicating no staining; 1 indicating 1–10%, 2 indicating 11–50%, and 3 indicating 51–100%. Then, multiplying the intensity score against the percentage staining score, we obtained a single scale with scores of 0, 1, 2, 3, 4, 6, and 9. A total score was calculated grouping scores 1–3 in total score 1, 4, and 6 in total score 2 and 9 in total score 3. Tissues with no staining in term of intensity and percentage of positive cells (total score=0) were scored as negative.

### TFF3 immunoassay

Serum TFF3 levels were measured by an in-house-specific ELISA, and each sample was analysed in duplicate. We used human TFF3 full-length recombinant protein (Abnova Corporation, Taipei City, Taiwan) for standard curve calibrator preparation, starting from a stock solution of 114 *μ*g ml^−1^. The stock solution was diluted in assay buffer (PBS 1% wt/vol bovine serum albumin, 0.05% Tween 20) to obtain calibrators ranging from 1.25 to 0.019 *μ*g ml^−1^. We used the assay buffer as zero calibrator. The mouse monoclonal antibody M01 clone 3D9 (Abnova Corporation) was used as capture antibody and for detection the mouse monoclonal antibody MAB 4407 (R&D Systems Inc., Minneapolis, MN, USA) previously biotinylated with a biotin solution (AH-BIOTIN-NHS, Biospa, Milano, Italy), following the manufacturer's protocol. Coating of Maxisorp flat-bottomed 96-well microtiter plates (Dako, Glostrup, Denmark) was done overnight at 4°C adding 100 *μ*l of 2.5 *μ*g ml^−1^ anti-human TFF3 M01 clone 3D9 in carbonate/bicarbonate buffer (Na_2_CO_3_ 0.0015 M, NaHCO_3_ 0.035 M) pH 9.6. Plates were subsequently washed four times with PBS 0.05% Tween 20 and blocked with 200 *μ*l of 3% (wt/vol) BSA in PBS 0.05% Tween 20 for 2 h at room temperature. After one wash, 100 *μ*l of standard curve and human sera diluted 1 : 5 in assay buffer were incubated for 2 h at room temperature.

After five washes, 100 *μ*l of biotinylated MAB 4407 antibody (diluted 1 : 100 in assay buffer) was added, and the plates were incubated for 2 h at room temperature. After five washes, 100 *μ*l of streptavidin peroxidase (Sigma-Aldrich Inc., St Louis, MO, USA) diluted to a concentration of 1 : 2000 with PBS 1% BSA was added to each well and incubated for 1 h, followed by five washes. The enzyme-catalyzed colour reaction was developed by the addition of 100 *μ*l of tetramethylbenzidine liquid substrate system (Sigma-Aldrich Inc.) to each well for 10 min. The colour development was stopped adding 100 *μ*l of 0.4 N sulphuric acid to each well and plates were read at 450 nm using an automatic plate reader (Spectramax 340 PC, Molecular Devices Corporation, Sunnyvale, CA, USA).

### CA125 serum levels measurements

Serum CA125 values in all samples were determined by the clinical laboratory at the Spedali Civili di Brescia, Italy, using the Architect CA125 II chemiluminescent two-step immunoassay kit (Abbott Diagnostics, Abbott Park, IL, USA) following the manufacturer's protocol.

### Statistical data analysis and clustering

Gene expression values were first analysed with a nonspecific filtering as previously reported (Bignotti *et al*, 2006). The comparison between EEC and NE samples was performed using the SAM algorithm (Tusher *et al*, 2001; Holger Schwender, 2007). Genes were considered of interest if the absolute value of the estimated fold change was equal or higher than 3, and if the fold discovery rate was smaller than 5%. A hierarchical clustering using 1−Pearson's correlation coefficient as distance matrix was performed to graphically show the results of the analysis.

Spearman's rank correlation was used to estimate the degree of association between microarray and qRT-PCR data for TFF3 gene. Exact Wilcoxon Mann–Whitney rank sum test was performed to estimate the difference in TFF3 immunohistochemical expression between EECs and NEs. Differences in TFF3 serum levels between the groups were calculated using ANOVA on log-transformed ELISA data. Spearman's rank correlation was used to estimate the degree of association between serum TFF3 and CA125 values. All the analyses were performed using the R (R Development Core Team, 2008) and Bioconductor software (Gentleman *et al*, 2004).

## Results

### Gene expression analysis and clustering of EEC and NE

**Figure 1:**
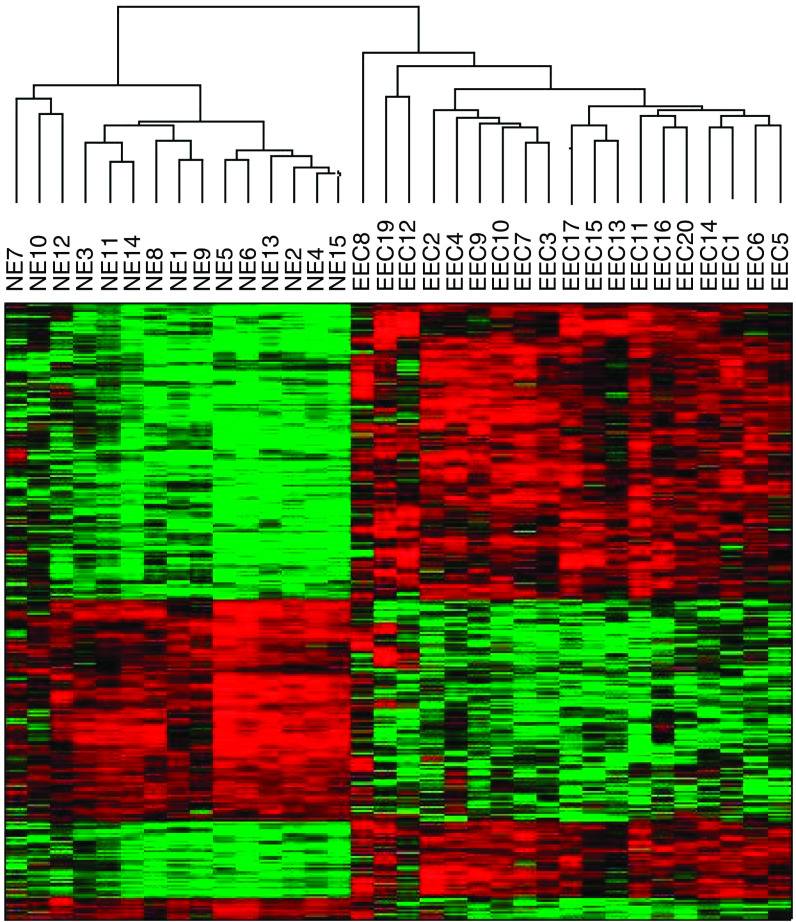
Dendrogram resulting from unsupervised cluster analysis differentiating EECs from NEs by gene expression profiling. The cluster is colour coded using red for upregulation, green for downregulation, and black for median expression.

Comprehensive gene expression profiles of 19 snap-frozen G3-EECs and 15 NEs were generated using high-density oligonucleotide microarrays. The unsupervised hierarchical sample cluster readily distinguished EECs from NEs showing two major branches. As shown in Figure 1, all 19 EECs were found to group together in the rightmost columns of the dendrogram, and similarly in the leftmost columns, all 15 NEs were found to cluster tightly together. After filtering out most ‘absent’ genes, the SAM analysis revealed a total of 922 probe sets showing >3-fold change and a fold discovery rate smaller than 5%. Out of 922 genes, 363 were found upregulated in EECs when compared when NEs (see Supplementary data), and among them, several genes encoding membrane and secreted proteins were found. Of great interest, owing to its secreted nature, TFF3 was the top differentially expressed gene in EECs, when compared with NE (fold change=21). The second profile was represented by 559 genes underexpressed in EECs and overexpressed in NEs (see Supplementary data).

### Validation of TFF3 gene expression by qRT-PCR

Quantitative real-time polymerase chain reaction technology was used to validate the different TFF3 mRNA expression in EEC, when compared with NE. Although data are not shown, we found qRT-PCR data for TFF3 to be significantly correlated to the microarray data (*P*<0.001; rs=0.85). Thus, qRT-PCR data suggest that most array probe sets are able to accurately measure the levels of the intended transcript within a complex mixture of transcripts.

### Validation of protein expression by immunohistochemical staining

**Figure 2:**
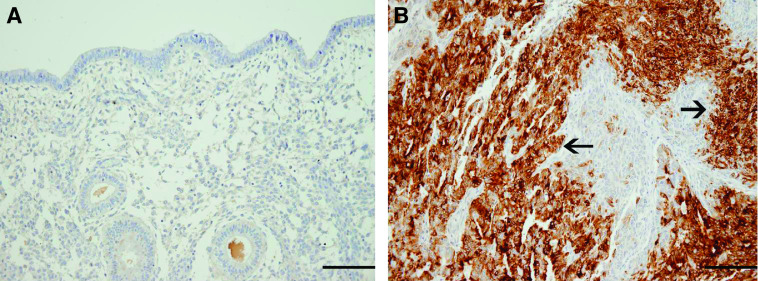
Representative immunohistochemical staining for TFF3. (**A**) Normal secretory endometrium showing no cytoplasmatic staining for TFF3 (total score=0, original magnification × 20). (**B**) Poorly differentiated endometrioid endometrial cancer displaying a strong cytoplasmatic positivity for TFF3 (total score=3, original magnification × 20). Trefoil factor 3 staining in EEC samples appeared to be diffuse, cytoplasmic, and restricted to the epithelial compartment, with no positivity in adjacent stromal cells. Arrows shows TFF3-positive epithelial cells *vs* the non-expressing stromal cells. Scale bar=50 *μ*m.

To confirm TFF3 gene expression results at the protein level, immunohistochemistry for TFF3 was carried out on 38 G3-EECs and 22 NEs. As shown in Table 1 and representatively displayed in Figure 2B, a positive cytoplasmatic staining for TFF3 was detected in 30 out of 38 (79%) EEC samples, whereas only 4 out of 22 (18%) NEs showed a weak immunoreactivity for TFF3 (Table 1 and Figure 2A). Tumour tissues showed markedly increased TFF3 positivity as compared with normal tissues (*P*<0.01). Trefoil factor 3 staining in EEC samples appeared to be diffuse, cytoplasmic, and restricted to the epithelial compartment, with no positivity in adjacent stromal cells. Trefoil factor 3 staining in tumour tissues appeared to be moderate/strong (total score 2/3) in 40% of cases (Table 1).

### TFF3 ELISA validation procedures

The assay sensitivity limit, defined as the concentration of TFF3 that can be distinguished from 0, was ∼5 ng ml^−1^ of recombinant TFF3 and detection was linear over a range of 20–1250 ng ml^−1^ (*r*^2^=0.99). We tested recombinant TFF3 diluted in assay buffer or ‘spiked’ into normal sera (at the same concentrations used for the standard curve), comparing the results using the ELISA assay. Recoveries were then calculated after subtraction of the serum endogenous concentration, and TFF3 detection sensitivity ranged from 88 to 100% in serum, when compared with assay buffer (data not shown). To evaluate the method reliability, four serum samples with different TFF3 levels, ranging from 500 to 1500 ng ml^−1^, were analysed eight times in the same ELISA experiment and on 6 different days. The intra-assay and inter-assay coefficients of variation (CV) were between 1.6–4.2 and between 4.1–8.2%, respectively.

### Serum TFF3 levels

**Figure 3:**
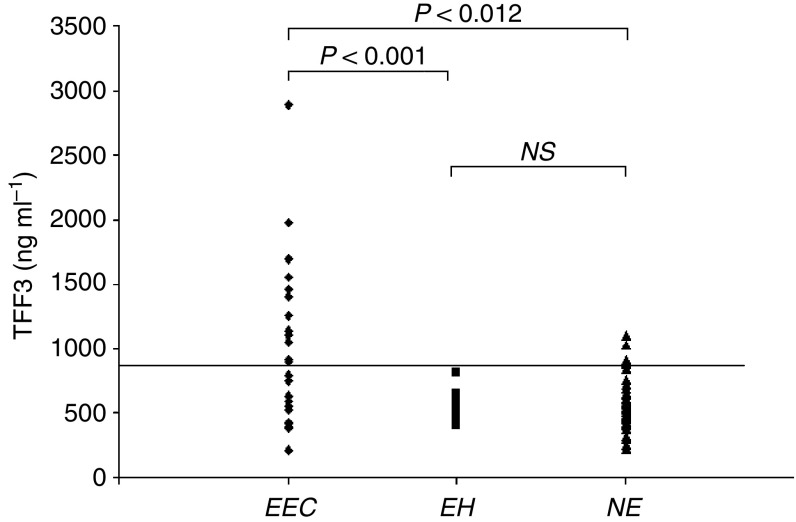
Trefoil factor 3 ELISA scatter plot in serum of poorly differentiated endometrioid endometrial cancer (EEC), endometrial hyperplasia (EH), and normal endometria (NE) patients. The cutoff value of 752 ng ml^−1^ is reported.

Table 2 shows the average TFF3 serum concentrations for patients with G3 endometrial cancer with endometrial hyperplasia and for healthy controls. Trefoil factor 3 serum concentration was significantly higher in patient with EEC compared with NE (*P*<0.001) and in EEC compared with EH (*P*=0.012). No difference was observed between serum TFF3 levels in EH, when compared with NE. Figure 3 displays TFF3 distribution for the three groups of patients. In this regard, to obtain a graphical plot of the sensitivity *vs* specificity of TFF3 levels for detecting and to better discriminate G3-EEC from controls, ROC curves were used. Using a cutoff value of 752 ng ml^−1^, the sensitivity and specificity of serum TFF3 for discriminating EEC from NE were 56 and 85%, respectively (Table 3), whereas setting a cutoff value of 587 ng ml^−1^, the sensitivity and specificity of serum TFF3 for detecting EEC compared with EH were 71 and 77%, respectively.

### Comparison of TFF3 and CA125 levels

We analysed CA125 serum levels in 25 G3-EEC patients and 42 controls tested with TFF3 ELISA. When the cutoff value was set at 35 U ml^−1^, the sensitivity of CA125 was 16% (4 of 25), whereas the specificity was 93% (39 of 42, Table 3). The sensitivity of CA125 in the detection of early-stage EECs was further decreased with only 10% of the stage I patients having a CA125>35 U ml^−1^. In the same group of patients, TFF3 assay was able to detect 60% (6 out of 10) of EEC at stage I. When the CA125 cutoff level was decreased at 20 U ml^−1^, as shown in Table 3, its sensitivity when compared with TFF3 remained significantly lower (i.e., 32 *vs* 56%, respectively). Because CA125 and TFF3 levels were not significantly correlated either in tumour patients or in negative controls, a combination of the two markers was analysed. The sensitivity and specificity of each marker and of the combination of the two are shown in Table 3. The combination of CA125 and TFF3 led to a sensitivity of 60% and a specificity of 67% considering all EEC stages.

## Discussion

Endometrial cancer is the most common gynaecologic malignancy in developed countries and it is generally considered a neoplasia with good prognosis. Indeed, most of the patients, due to the early declaration of the disease by vaginal bleeding, are diagnosed at an early stage and with Type I EC. Nevertheless, up to 35% of EC patients may be diagnosed with biologically aggressive Type II tumours, with G3-EEC accounting for the majority of the cases (Bokhman, 1983). For several of these patients, the prognosis remains poor, regardless of their treatment with gold standard therapies including surgery, adjuvant radiation, and/or chemotherapy. Furthermore, few EC markers are currently available to monitor the effects of adjuvant therapy or to predict early tumour recurrence. In this regard, although CA125 is commonly used in the clinic for these purposes, it is endowed with low sensitivity and specificity (Sood *et al*, 1997; Powell *et al*, 2005).

In the present investigation, with the aim to discover new diagnostic molecular markers for G3-ECC, we have analysed the gene expression profile of G3-EECs, the most common Type II uterine cancer. A genome wide examination of this aggressive tumour variant with the more comprehensive Affymetrix chip currently available (i.e., HG-U133 plus 2.0 covering 47 000 human transcripts and variants) has provided evidence that G3-ECC genetic fingerprints can be clearly distinguished from those of NE. Indeed, we detected 922 differentially expressed genes, whose average change in expression level between the two groups was at least threefold. At the top of overexpressed genes in EECs compared with NEs, with a fold change of 21, TFF3 was found.

Trefoil factor 3 belongs to a family of small, compact peptides containing one or two trefoil domains, consisting of 42 to 43 amino acids with six cysteine residues forming three disulphide bonds, giving the characteristic three-leafed structure (Thim, 1997). Trefoil factor 3 was recognised for the first time in rat intestine, and it has been shown to be primarily expressed and secreted onto the intestinal surface by goblet cells of the human small and large intestinal mucosa (Suemori *et al*, 1991; Podolsky *et al*, 1993). The main reported TFF3 role in the gastrointestinal tract involves the reconstitution of the mucosal barrier to protect the epithelial layer against environmental injury induced by ulceration and inflammation, increasing the rate of epithelial migration into the wound (Dignass *et al*, 1994; Xian *et al*, 1999).

Trefoil factor 3 has been shown to be expressed in several normal tissues including hypothalamus/pituitary, breast, conjunctiva, and salivary gland (Probst *et al*, 1996; Poulsom *et al*, 1997; Langer *et al*, 1999; Devine *et al*, 2000). Abnormally elevated levels of TFF3 have been documented in breast, pancreatic, gastric, and prostate carcinomas (Theisinger *et al*, 1996; Terris *et al*, 2002; Yamachika *et al*, 2002; Garraway *et al*, 2004). Importantly, overexpression of TFF3 has been reported to be prognostically important in several of these cancers (Yamachika *et al*, 2002; Yio *et al*, 2004). Nevertheless, little is known about whether TFF3 directly contributes to the malignant behaviour of cancer cells. In this regard, TFF3 has been shown to regulate cancer progression by increasing tumour metastasis acting as anti-apoptotic, scattering, pro-invasive, and angiogenic agent on cancer cells (Taupin *et al*, 2000; Emami *et al*, 2001; Rodrigues *et al*, 2003). Trefoil factor 3 expression in the human uterus has been analysed by several investigators with conflicting results. For instance, Wiede *et al* (2001) reported low TFF3 mRNA levels by qRT-PCR and no detectable TFF3 protein expression in the NE by Western blot analysis. Accordingly, Madsen *et al* (2007) found few endometrial cells positively stained for TFF3 by immunohistochemistry. In contrast, Borthwick *et al* (2003) documented different TFF3 transcript levels during the phases of the menstrual cycle, with a major TFF3 expression in proliferative compared with secretory endometrium, suggesting its role in regeneration of the human endometrium following menstruation.

According to our microarray results, TFF3 mRNA was found to be consistently upregulated in the majority of G3-EEC specimens as compared with NE tissues. Gene expression results have been validated successfully on the same set of samples by qRT-PCR, confirming TFF3 gene expression profiling data. Moreover, we demonstrated TFF3 overexpression in G3-EEC tissues by immunohistochemical staining, providing the first evidence of TFF3 protein upregulation in EC. In agreement with previous reports, TFF3 was found negative or focally positive by immunostaining in NE (Wiede *et al*, 2001; Madsen *et al*, 2007). In contrast to the result of Borthwick *et al*, however, we did not observe significant differences in TFF3 mRNA or protein expression between the different menstrual cycle phases in NE (data not shown).

Importantly, as TFF3 is a secreted peptide, it may represent a novel, potentially useful diagnostic biomarker in G3-EEC patients. Consistent with this view, TFF3 levels were measured by a specific in-house ELISA developed in our laboratory in 25 G3-EEC patients treated at our Institution. Our results showed a significantly higher TFF3 serum level in EEC patients when compared with healthy women or patients harbouring endometrial hyperplasia. More importantly, TFF3 serum levels showed higher sensitivity in the detection of patients harbouring G3-EEC when compared with CA125 (cutoff=35 U ml^−1^). Furthermore, similar results were found when CA125 was compared with TFF3 levels at a cutoff value of 20 U ml^−1^, reported by several investigators to be more appropriate for preoperative evaluation and post-operative surveillance of EC patients (Sood *et al*, 1997; Kurihara *et al*, 1998). Indeed, even at this lower CA125 setting, TFF3 remained a more sensitive indicator of tumour presence than CA125. Finally, when we analysed the combined ability of CA125 and TFF3 serum markers in detecting endometrial cancer, we found no significant improvement when compared with TFF3 alone.

In conclusion, several novel tumour-restricted markers have been identified through our genome-wide analysis of G3-EEC. The identification of TFF3 as a novel diagnostic biomarker endowed with a sensitivity and specificity superior to that of CA125 in the preoperative evaluation of G3-EEC patients, as demonstrated in this pilot work, may support the design of prospective studies evaluating the potential of TFF3 as a new tool for preoperative evaluation and post-operative surveillance of EC patients.

## Figures and Tables

**Table 1:** TFF3 IHC results

**Characteristic**	**EEC**	**NE**
*n*	38	22
		
*Stage*		
IA	1	
IB	8	
IC	9	
IIA	2	
IIB	9	
IIIA	2	
IIIC	5	
IV	2	
		
*TFF3 staining (total score)* a		
0	8 (21%)	18 (82%)
1	15 (39%)	4 (18%)
2	8 (21%)	0
3	7 (19%)	0

EEC=endometrioid endometrial carcinomas; NE=normal endometrial cells; TFF3=trefoil factor 3.

a TFF3 staining indicates scoring method was based on the intensity of the staining and on the percentage of tumour cells stained as described in the Materials and Methods section.

**Table 2:** TFF3 and CA125 quantification in patient sera

		**CA125 (U ml^−1^)**		**TFF3 (ng ml^−1^)**	
**Patient group**	***n***	**Range**	**Mean**	**Range**	**Mean**
EEC	25	4–853	46	213–2890	955
EH	13	9–61	20	409–813	523
NE	47	1–96	16	226–1101	556

EEC=endometrioid endometrial carcinomas; EH=endometrial hyperplasia cells; NE=normal endometrial cells; TFF3=trefoil factor 3.

**Table 3:** Sensitivity and specificity of CA125, TFF3 and the combination of both markers

				**Combination (%)**
**Cutoff**	**CA125 (%)** **20 U ml^−1^**	**CA125 (%)** **35 U ml^−1^**	**TFF3 (%)** **752 ng ml^−1^**	**20 U ml^−1^ and 752 ng ml^−1^**
Sensitivity^a^	32	16	56	60
Specificity^b^	81	93	85	67

TFF3=trefoil factor 3.